# Improving Classification Performance in Dendritic Neuron Models through Practical Initialization Strategies

**DOI:** 10.3390/s24061729

**Published:** 2024-03-07

**Authors:** Xiaohao Wen, Mengchu Zhou, Aiiad Albeshri, Lukui Huang, Xudong Luo, Dan Ning

**Affiliations:** 1 Teachers College for Vocational and Technical Education, Guangxi Normal University, Guilin 541001, China; wenxiaohao@gxnu.edu.cn (X.W.); luoxudong@gxnu.edu.cn (X.L.); ningdan@stu.gxnu.edu.cn (D.N.); 2 Faculty of Innovation Engineering, Macau University of Science and Technology, Macau 999078, China; 3 Department of Electrical and Computer Engineering, New Jersey Institute of Technology, Newark, NJ 07102, USA; 4 Department of Computer Science, King Abdulaziz University, Jeddah 21481, Saudi Arabia; aaalbeshri@kau.edu.sa; 5 School of Accounting and Audit, Guangxi University of Finance and Economics, Nanning 530031, China; lukui-hua59@tbs.tu.ac.th

**Keywords:** dendritic neuron model, initialization methods, deep learning, neural networks

## Abstract

A dendritic neuron model (DNM) is a deep neural network model with a unique dendritic tree structure and activation function. Effective initialization of its model parameters is crucial for its learning performance. This work proposes a novel initialization method specifically designed to improve the performance of DNM in classifying high-dimensional data, notable for its simplicity, speed, and straightforward implementation. Extensive experiments on benchmark datasets show that the proposed method outperforms traditional and recent initialization methods, particularly in datasets consisting of high-dimensional data. In addition, valuable insights into the behavior of DNM during training and the impact of initialization on its learning performance are provided. This research contributes to the understanding of the initialization problem in deep learning and provides insights into the development of more effective initialization methods for other types of neural network models. The proposed initialization method can serve as a reference for future research on initialization techniques in deep learning.

## 1. Introduction

Deep learning has achieved remarkable success in a variety of machine learning tasks, such as image recognition, speech recognition, and natural language processing [1,2,3]. One of the key reasons for this success is the ability of deep neural networks to automatically extract complex features from raw data collected from various sensors and other means [4]. However, their training is a non-trivial task. One of the main challenges is the proper initialization of their network weights. It has a significant impact on their learning performance. Poor initialization can lead to gradient vanishing or explosion, which can severely impede a learning process [5]. Gradient vanishing occurs when the gradients propagated through the network become too small, and the weights are not updated effectively. Gradient explosion, on the other hand, occurs when the gradients become too large, causing the weights to update too much and destabilizing a learning process.

The initialization issue for deep neural networks has been widely studied in the literature, and various initialization methods have been proposed to improve their learning performance. Random initialization is one of the most commonly used initialization methods. In this method, their weights are randomly initialized from a uniform or Gaussian distribution. However, this method has several limitations, including the inability to take into account network structures and the lack of control over the magnitude of weights. Moreover, random initialization may lead to gradient vanishing or explosion problems in training. Pre-training is another initialization method that has been widely used in deep learning. This method involves training a shallow network layer by layer and using the learned weights to initialize deep networks [6]. While pre-training can improve their learning performance, it is computationally expensive and may not be effective for certain types of neural network models. Other advanced initialization methods have been proposed in recent years to address the limitations of traditional methods. The Xavier initialization [5] and He initialization [7] are two widely used ones, which take into account network structures and activation functions. The former sets the variance of weights based on the number of input and output neurons, while the latter sets the variance based on the number of input neurons. Abbe et al. [8] introduce an approach to control the overfitting problem in neural networks by aligning the initialization process with the target function. However, the issue of gradient vanishing or explosion in deep network training may still occur.

In addition to the above methods, there have been several studies on initialization methods for special neural network models, such as convolutional neural networks (CNNs) and recurrent neural networks (RNNs). For example, He et al. [9] presented a variant of the He initialization for CNNs, taking into account the spatial dimensions of the filters. Mayer et al. [10] utilized the input sequences as a fractal dimension to optimize the recurrent neural network initialization. Humbird et al. [11] proposed initializing deep feedforward neural networks with decision trees for performance enhancement. Gabrielli et al. [12] presented an actuarial loss reserving technique, taking into account both claim counts and claim amounts.

However, these initialization methods may not be directly applicable to a dendritic neuron model (DNM), which has a unique neuron structure and activation function. DNM is a deep neural network model with a dendritic tree structure, which enables it to perform complex logic operations and approximate functions more accurately than traditional neuron models [13,14,15]. Its dendritic structure provides a rich set of features that can be leveraged to enhance its learning performance. However, the complex dendritic structure of DNM also poses new challenges. In particular, when processing high-dimensional data, DNMs are more prone to issues of gradient vanishing/exploding. While great research efforts have been devoted to improving the learning performance of original DNM [16,17,18,19,20], the challenges related to gradients and computational costs when handling high-dimensional data remain to be addressed.

Therefore, the objective of this work is to investigate initialization methods with a special focus on gradient vanishing related to handling high-dimensional data, and to evaluate their effectiveness in improving its learning performance. This work aims to make the following novel contributions:

1. A novel but simple initialization method for DNM, which takes into account its unique characteristics, such as its dendritic structures and activation functions. It can effectively initialize the weights of DNM for its optimal learning performance.
2. Performing extensive experiments on benchmark datasets to evaluate the effectiveness of the proposed initialization method and comparing it with traditional and advanced initialization methods, in order to provide a comprehensive understanding of the impact of different initialization techniques on the learning performance of the DNM.

This research contributes to the understanding of the initialization problem in deep learning, and provides valuable insights into the development of more effective initialization methods for other types of models.

The remainder of this paper is organized as follows. Section 1 introduces the background and related work of DNM. Section 2 reviews the model derivation and analysis of DNM. Section 3 presents the proposed initialization method for DNM. Section 4 shows experimental results and their discussions. Finally, Section 5 concludes the paper and outlines future research directions.

## 2. Dendritic Neuron Model

Contemporary artificial neural networks (ANNs) are intricately designed, utilizing a myriad of simplistic units to build their architecture. The prevalent model employed within ANN is the feedforward multilayer perceptron (MLP), deriving its foundational concepts from the classic McCulloch–Pitts neuron [21,22,23]. Cutting-edge research has revealed that individual neurons—when factoring in the nonlinear dynamic processes occurring in synapses and the adaptability of dendritic structures—can match the computational prowess exhibited by networks composed of multiple neurons [24,25,26]. Such revelations have guided the conceptualization of DNMs as delineated in. They demonstrate a strong ability to deal with nonlinear problems [27,28].

**Figure 1:**
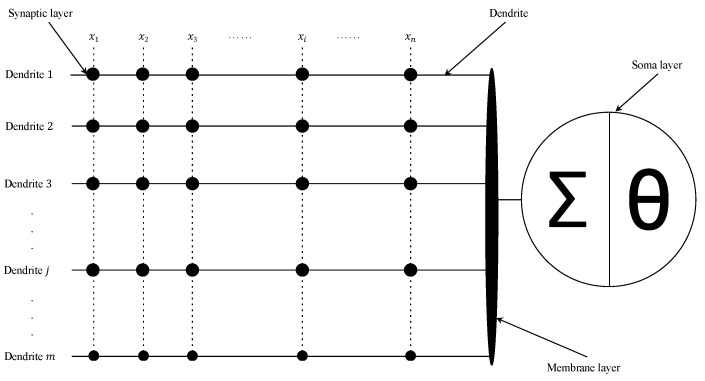
An *m*-dendrite and *n*-input dendritic neuron model [27,28,29,30].

Mirroring the functions of biological neurons, a DNM is stratified into four layers: synaptic layer, dendrite layer, membrane layer, and soma layer. The initial layer encodes external inputs into neural signals through a sigmoid activation function. These signals then converge along dendritic branches, with each dendrite multiplying the outputs from the synaptic juncture. The subsequent membranous layer aggregates these multiplicative outputs before forwarding them to the somatic layer, employing another sigmoid function to culminate the neural computation process. DNMs are engineered to mimic the structural intricacies and signal propagation mechanisms observed in natural neurons, characterized by their straightforward architecture, ease of implementation, and notable clarity in interpretability. By emulating biological paradigms, DNMs forge a meaningful nexus with artificial intelligence, providing a platform to decipher the complexities of biological neural networks, which merits in-depth exploration. Its detailed structure is illustrated in Figure 1, as described next.

Recent studies have significantly advanced our understanding of DNMs, exploring their applicability across a wide range of fields. Ji et al. [14] introduced an approximate logic neuron model with a dendritic structure, which was later trained using states of matter search algorithm [29]. Gao et al. [30] presented a DNM with effective learning algorithms that excel in classification, approximation, and prediction tasks. He et al. [31] employed a DNM for financial time series prediction, harnessing seasonal-trend decomposition to enhance its predictive capabilities.

Furthermore, the work of Xu et al. [32] and Gao et al. [33] not only illustrated innovative methodologies in the training of DNMs—such as the use of information feedback-enhanced differential evolution and the creation of a fully complex-valued DNM—but also catalyzed a series of subsequent studies that leveraged these advanced techniques to push the boundaries of what DNMs could achieve in various fields of application [34,35,36]. Yilmaz and Yolcu [37] leveraged a modified particle swarm optimizer to train DNM for time-series forecasting, while Egrioglu et al. [38] introduced a recurrent DNM aimed at the same application.

The practical applications of DNMs are notably diverse, as demonstrated by Tang et al. [39], who adopted a dendritic neural model for predicting stock price index movement, and Al-qaness et al. [40], who utilized an optimized DNM for wind power forecasting. A comprehensive survey by Ji et al. [41] provides an in-depth look at the mechanisms, algorithms, and practical applications of DNMs, highlighting the extensive research and development in this field. Furthermore, Yu et al. [42] improved DNM with a dynamic scale-free network-based differential evolution, and Ji et al. [43] proposed a competitive decomposition-based multiobjective architecture search for DNM. The latest network of dendritic neurons by Peng et al. [44] showcases the ongoing efforts to enhance the functionality and efficiency of DNMs.

### 2.1. Synaptic Layer

The synaptic layer serves as the communication hub within a neuron, where signals are transmitted and received. It plays a crucial role in regulating and controlling the activities of neurons. Synapses can be classified into excitatory ones, which excite a postsynaptic neuron, and inhibitory ones, which inhibit it. When the accumulated neurotransmitters released from the presynaptic neuron exceed a certain threshold, an action potential is generated. This process determines whether a synapse is excitatory or inhibitory. We use a sigmoid function to represent the connection state between the *i*-th (i=1,2,...,N) synaptic input and the *j*-th (j=1,2,...,M) synaptic layer:

(1) $$s_{ij}=\sigma \left(w_{ij}x_{i}-\theta_{ij}\right)=\frac{1}{1+e^{-k(w_{ij}x_{i}-\theta_{ij})}},$$

where $s_{ij}$ denotes the output from the *i*-th synaptic input to the *j*-th synaptic layer, σ represents the sigmoid function, and $x_{i}\in \left[0,1\right]$ indicates the input of a synapse. *k* is a positive scaling factor. The connection weight, $w_{ij}$, and threshold, $\theta_{ij}$, are parameters to be learned.

### 2.2. Dendrite Layer

Within a branch, a dendrite conducts multiplicative functions across its array of synaptic junctions. This mechanism encapsulates the nonlinear interplay among synaptic communications that unfold along individual dendritic pathways—effectively realized through multiplication. The transference of signals within dendrites adheres to a binary schema, with the inputs and outputs being restricted to the values of 1 or 0. Consequently, the synaptic interconnections along the dendritic branches fundamentally equate to logical AND operations:

(2) $$d_{j}=\prod_{i=1}^{N}s_{ij},$$

where $d_{j}$ represents the output of the *j*-th dendrite.

### 2.3. Membrane Layer

The membrane layer aggregates the signals from all dendrites. The inputs received from *M* dendritic branches are combined, analogous to a logical OR operation. The output of the membrane layer *m* is expressed as follows:

(3) $$m=\sum_{j=1}^{M}d_{j},$$

The resulting output is then transmitted to the soma layer.

### 2.4. Soma Layer

The soma layer generates the final output. When the output from the membrane layer surpasses a threshold, the neuron elicits a spike. A sigmoid function is used as follows:

(4) $$o=\sigma_{s}\left(m-\theta_{s}\right)=\frac{1}{1+e^{-k_{s}\left(m-\theta_{s}\right)}},$$

where $\theta_{s}\in \left[0,1\right]$ represents the threshold of the soma layer and $k_{s}$ is a positive scaling factor.

### 2.5. Learning Algorithm

Given that DNM operates as a feedforward architecture and incorporates differentiable functions, it is well-suited for employing the error back-propagation algorithm (BP) for learning. The BP algorithm systematically updates the parameters $w_{ij}$ and $\theta_{ij}$ by leveraging the learning rate and gradient descent to minimize the disparity between the actual output *o* and the target output $\hat{o}$. The squared error, representing the discrepancy between *o* and $\hat{o}$, is quantified as follows:

(5) $$E=\frac{1}{2}{\left(\hat{o}-o\right)}^{2},$$

In DNM, *E* is minimized by modifying the connection parameters in the negative gradient direction in an iterative process. Therefore, differential changes of these connection parameters need to be collected, i.e.,

(6) $$\Delta w_{ij}=-\eta \frac{\partial E}{\partial w_{ij}},$$

(7) $$\Delta \theta_{ij}=-\eta \frac{\partial E}{\partial \theta_{ij}},$$

where η is a learning rate whose values range from 0.01 to 0.1. BP updates $w_{ij}$ and $\theta_{ij}$ according to the following:

(8) $$w_{ij}\left(t+1\right)=w_{ij}\left(t\right)+\Delta w_{ij}$$

(9) $$\theta_{ij}\left(t+1\right)=\theta_{ij}\left(t\right)+\Delta \theta_{ij}$$

## 3. Model Derivation and Analysis

### 3.1. Gradient Vanishing

To simplify our analysis work, we assume that DNM only has one dendritic layer and one output neuron. The dendritic layer has *n* synaptic inputs with corresponding synaptic weights, $w_{i}$, and threshold values, $\theta_{i}$. The output neuron has a sigmoid activation function, characterized by a weight, $w_{s}$, and a threshold, $\theta_{s}$.

The output of the *i*-th synapse can be simplified from (1) into the following:

(10) $$s_{i}=\frac{1}{1+e^{-k(w_{i}x_{i}-\theta_{i})}},$$

where $x_{i}\in \left[0,1\right]$ is the input of the synapse, and *k* is a positive constant. The output of the dendritic layer can be calculated as follows:

(11) $$d=\prod_{i=1}^{n}s_{i}$$

The output of the output neuron is as follows:

(12) $$o=\frac{1}{1+e^{-k_{s}\left(w_{d}-\theta_{s}\right)}},$$

where $w_{d}=d-\theta_{s}$, $k_{s}$, and $\theta_{s}$ are positive constants.

The gradient of the output neuron, with respect to the weight, $w_{i}$, can be calculated as follows:

(13) $$\frac{\partial o}{\partial w_{i}}=\frac{\partial o}{\partial d}\frac{\partial d}{\partial s_{i}}\frac{\partial s_{i}}{\partial w_{i}}$$

The partial derivative $\frac{\partial o}{\partial d}$ can be expressed as follows:

(14) $$\frac{\partial o}{\partial d}=\frac{k_{s}e^{-k_{s}\left(w_{d}-\theta_{s}\right)}}{{\left(1+e^{-k_{s}\left(w_{d}-\theta_{s}\right)}\right)}^{2}}$$

The partial derivative $\frac{\partial d}{\partial s_{i}}$ can be expressed as follows:

(15) $$\frac{\partial d}{\partial s_{i}}=\prod_{j\neq i}^{n}s_{j}$$

The partial derivative $\frac{\partial s_{i}}{\partial w_{i}}$ can be expressed as follows:

(16) $$\frac{\partial s_{i}}{\partial w_{i}}=kx_{i}e^{-k(w_{i}x_{i}-\theta_{i})}/{\left(1+e^{-k(w_{i}x_{i}-\theta_{i})}\right)}^{2}$$

Therefore, the gradient of the output neuron with respect to weight, $w_{i}$, can be calculated as follows:

(17) $$\frac{\partial o}{\partial w_{i}}=\frac{k_{s}e^{-k_{s}\left(w_{d}-\theta_{s}\right)}}{{\left(1+e^{-k_{s}\left(w_{d}-\theta_{s}\right)}\right)}^{2}}\prod_{j\neq i}^{n}s_{j}\frac{kx_{i}e^{-k(w_{i}x_{i}-\theta_{i})}}{{\left(1+e^{-k(w_{i}x_{i}-\theta_{i})}\right)}^{2}}$$

We can see that the gradient calculation in DNM with multiplication in the dendritic layer involves a product of all the output values, which is multiplied with the partial derivative of the *i*th output value. As the number of inputs increases, the product of all the output values can become very small, leading to gradient vanishing. This is the reason why gradient vanishing occurs when training a dendritic neuron model with multiplication in the dendritic layer. Therefore, it can be concluded that DNM training is prone to gradient vanishing.

### 3.2. Gradient Vanishing with Different Initialization Methods

In this section, we analyze the gradient vanishing issue for different weight initialization methods in the context of DNM. Specifically, we calculate the expected values, mean values, and variances of the outputs after passing through the synaptic layer for each initialization method. By relating these values to the gradient vanishing discussion, we can assess their susceptibility to the gradient vanishing issue.

For each initialization method, let $s_{i}$ denote the output of the *i*-th synapse, and recall the expression for $s_{i}$:

(18) $$s_{i}=\frac{1}{1+e^{-k(w_{i}x_{i}-\theta_{i})}}$$

We assume that input $x_{i}$ follows a uniform distribution over the interval [0,1].

With the expected values of $s_{i}$ for each initialization method, we can assess the gradient vanishing issue by referring to the methodology used in the gradient vanishing discussion. Additionally, we provide a graphical representation of the gradient expectation and variance for each initialization method, as shown in the provided Python code.

#### 3.2.1. Random Initialization

In this case, we assume that the synaptic weight, $w_{i}$, and threshold value, $\theta_{i}$, are initialized randomly over the interval (−1,1), and $x_{i}$ is in the interval (0,1). The expected value of $s_{i}$ can be derived as follows:

(19) $$E\left[s_{i}\right]=\int_{0}^{1}\int_{-1}^{1}\int_{-1}^{1}\frac{1}{1+e^{-k(w_{i}x_{i}-\theta_{i})}}\cdot \frac{1}{4}dw_{i}d\theta_{i}dx_{i}$$

#### 3.2.2. Normal Initialization

In this case, we assume that the synaptic weight, $w_{i}$, and threshold value, $\theta_{i}$, are initialized with a Gaussian distribution with a mean of 0 and a standard deviation, σ, and $x_{i}$ is in the interval (0,1). The expected value of $s_{i}$ is as follows:

(20) $$E\left[s_{i}\right]=\int_{0}^{1}\int_{-\infty }^{\infty }\int_{-\infty }^{\infty }\frac{1}{1+e^{-k(wx-\theta )}}\cdot \frac{1}{2\pi \sigma^{2}}e^{-\frac{w^{2}+\theta^{2}}{2\sigma^{2}}}dwd\theta dx$$

#### 3.2.3. He Initialization

In this method, the synaptic weight, $w_{i}$, and threshold value, $\theta_{i}$, are initialized with a Gaussian distribution with a mean of 0 and standard deviation, $\sqrt{\frac{2}{n_{in}}}$, where $n_{in}$ is the number of input neurons, and $x_{i}$ is in the interval (0,1). The expected value of $s_{i}$ is as follows:

(21) $$E\left[s_{i}\right]=\int_{0}^{1}\int_{-\infty }^{\infty }\int_{-\infty }^{\infty }\frac{1}{1+e^{-k(wx-\theta )}}\cdot \frac{1}{2\pi (\frac{2}{n_{in}})}e^{-\frac{n_{in}\left(w^{2}+\theta^{2}\right)}{4}}dwd\theta dx$$

#### 3.2.4. Xavier Initialization

In this method, the synaptic weight, $w_{i}$, and threshold value, $\theta_{i}$, are initialized with a Gaussian distribution with a mean of 0 and standard deviation $\sqrt{\frac{1}{n_{in}}}$, where $n_{in}$ is the number of input neurons, and $x_{i}$ is in the interval (0,1). The expected value of $s_{i}$ is as follows:

(22) $$\begin{array}{c} E\left[s_{i}\right]=\int_{0}^{1}\int_{-\infty }^{\infty }\int_{-\infty }^{\infty }\frac{1}{1+e^{-k(wx-\theta )}}\cdot \frac{1}{2\pi (\frac{1}{n_{in}})}e^{-\frac{n_{in}\left(w^{2}+\theta^{2}\right)}{2}}dwd\theta dx \end{array}$$

The expected values of $s_{i}$ for each initialization method are summarized in Table 1. By comparing the expected values, we can evaluate the performance of different initialization methods in mitigating the gradient vanishing issue. Furthermore, the graphical results provided in the Python code can help one visualize the effects of these initialization methods on the gradient expectation and variance.

### 3.3. Gradient Vanishing after Dendrite Layer

After we calculate the expected values, mean values, and variances of the outputs after passing through the synaptic layer, we can analyze the possibility of gradient vanishing after passing through the dendrite layer. Recall the expression of the output of the dendrite layer. We can study the gradient vanishing problem by looking at the variance of product *d*. If it is small, then the gradient of the output neuron with respect to weight, $w_{i}$, would also be small, leading to gradient vanishing.

#### 3.3.1. Random Initialization

The product of all output values, $\prod_{j\neq i}^{n}s_{j}$, can be highly variable due to the random nature of the synaptic weights and threshold values. This variability increases the chances of the product becoming very small, leading to gradient vanishing.

The expected value of the product of the output values is as follows:

(23) $$E\left[\prod_{j\neq i}^{n}s_{j}\right]={\left(\int_{0}^{1}\int_{-1}^{1}\int_{-1}^{1}\frac{1}{1+e^{-k(wx-\theta )}}\cdot \frac{1}{4}dwd\theta dx\right)}^{n-1}$$

As the number of inputs, *n*, increases, it becomes smaller, thus increasing the likelihood of gradient vanishing.

#### 3.3.2. Normal Initialization

With normal initialization, the product of all output values, $\prod_{j\neq i}^{n}s_{j}$, is subject to the variability introduced by the Gaussian distribution of synaptic weights and threshold values. This variability could cause the product of the output values to become very small, leading to gradient vanishing.

The expected value of the product of the output values is as follows:

(24) $$E\left[\prod_{j\neq i}^{n}s_{j}\right]=\left(\int_{0}^{1}\int_{-\infty }^{\infty }\int_{-\infty }^{\infty }\left.\frac{1}{1+e^{-k(wx-\theta )}}\cdot \frac{1}{2\pi \sigma^{2}}e^{-\frac{w^{2}+\theta^{2}}{2\sigma^{2}}}\right.\right.{\left.dwd\theta dx\right)}^{n-1}$$

As the number of inputs, *n*, increases, it becomes smaller, making the gradient vanishing problem more likely.

#### 3.3.3. He Initialization

With He initialization, the product of all output values, $\prod_{j\neq i}^{n}s_{j}$, is subject to the variability introduced by the Gaussian distribution of synaptic weights and threshold values with standard deviation, $\sqrt{\frac{2}{n_{in}}}$. This variability could cause the product of the output values to become very small, leading to gradient vanishing.

The expected value of the product of the output values is as follows:

(25) $$E\left[\prod_{j\neq i}^{n}s_{j}\right]=\left(\int_{0}^{1}\int_{-\infty }^{\infty }\int_{-\infty }^{\infty }\left.\frac{1}{1+e^{-k(wx-\theta )}}\cdot \frac{1}{2\pi (\frac{2}{n_{in}})}\right.\right.{\left.e^{-\frac{n_{in}\left(w^{2}+\theta^{2}\right)}{4}}dwd\theta dx\right)}^{n-1}$$

As the number of inputs, *n*, increases, it becomes smaller, causing a gradient vanishing problem.

#### 3.3.4. Xavier Initialization

With Xavier initialization, the product of all output values, $\prod_{j\neq i}^{n}s_{j}$, is subject to the variability introduced by the Gaussian distribution of synaptic weights and threshold values with standard deviation, $\sqrt{\frac{1}{n_{in}}}$. This variability could cause the product of the output values to become very small, leading to gradient vanishing.

The expected value of the product of the output values is as follows:

(26) $$E\left[\prod_{j\neq i}^{n}s_{j}\right]=\left(\int_{0}^{1}\int_{-\infty }^{\infty }\int_{-\infty }^{\infty }\left.\frac{1}{1+e^{-k(wx-\theta )}}\cdot \frac{1}{2\pi (\frac{1}{n_{in}})}\right.\right.{\left.e^{-\frac{n_{in}\left(w^{2}+\theta^{2}\right)}{2}}dwd\theta dx\right)}^{n-1}$$

As the number of inputs, *n*, increases, the expected value of the product of the output values can become very small, making the gradient vanishing problem more likely.

### 3.4. Proposed Initialization Method

In order to mitigate the gradient vanishing problem in training a DNM with multiplication in the dendritic layer, we propose a new initialization method called non-negative initialization. For the *i*-th synapse, we initialize weight, $w_{i}$, as a random non-negative value, for example, by following a uniform distribution within the range of [0,b], where *b* is a positive number. In the following, we will provide a mathematical analysis to justify why this strategy can mitigate the gradient vanishing issue.

As previously discussed, the gradient of the output neuron with respect to the synaptic weight, $w_{i}$, involves the product of all the output values. When these output values become too small, the product term can quickly approach zero, leading to the vanishing gradient issue.

Now, consider the initialization of $w_{i}$ within the range of [0,b]. Denote $f_{i}=w_{i}x_{i}-\theta_{i}$. If $w_{i}$ and $x_{i}$ are both uniformly distributed within their respective ranges, the expected value of $f_{i}$ is then $\frac{b}{2}\times \frac{1}{2}-\theta_{i}=\frac{b}{4}-\theta_{i}$.

Consequently, the expected value of the output from the *i*th synapse, given by $s_{i}=\frac{1}{1+e^{-kf_{i}}}$, is as follows:

(27) $$E\left[s_{i}\right]=\frac{1}{1+e^{-k(\frac{b}{4}-\theta_{i})}}$$

If we choose $\theta_{i}=\frac{b}{4}$, then $E\left[s_{i}\right]=\frac{1}{1+e^{0}}=\frac{1}{2}$, which is well-centered in its possible output range of [0,1]. This means that, on average, $s_{i}$ is neither too small nor too large, thereby avoiding the problem of vanishing or exploding gradients.

Furthermore, since the synaptic weights, $w_{i}$, are initialized as non-negative values, the product term in the gradient formula, $\prod_{j\neq i}^{n}s_{j}$, is ensured to be non-negative. This further helps mitigate the gradient vanishing issue.

### 3.5. Non-Negative Initialization: Detailed Analysis

In this section, we provide a more detailed analysis of the non-negative initialization method, incorporating the expected values, mean values, and variances. We aim to provide a thorough and well-founded explanation of our proposed method.

#### 3.5.1. Analysis of Synaptic Outputs

Considering the non-negative initialization method—the weight, $w_{i}$, is initialized within the range of [0,b]. Assume that $w_{i}$ follows a uniform distribution in this range. Therefore, the expected value of $w_{i}$ is as follows:

(28) $$E\left[w_{i}\right]=\frac{b}{2}$$

Assume that $x_{i}$ also follows a uniform distribution in the range of [0,1]. Then, the expected value of $x_{i}$ is as follows:

(29) $$E\left[x_{i}\right]=\frac{1}{2}$$

Thus, the expected value of the product $w_{i}x_{i}$ can be calculated as follows:

(30) $$E\left[w_{i}x_{i}\right]=E\left[w_{i}\right]E\left[x_{i}\right]=\frac{b}{4}$$

As a result, the expected value of the synaptic output, $s_{i}$, is as follows:

(31) $$E\left[s_{i}\right]=\frac{1}{1+e^{-k(E\left[w_{i}x_{i}\right]-\theta_{i})}}$$

The mean value and variance of $s_{i}$ can be derived from the distribution of $s_{i}$. Since $w_{i}$ and $x_{i}$ follow uniform distributions, their product, $w_{i}x_{i}$, has a mean value of $\frac{b}{4}$ and a variance of $\frac{b^{2}}{48}$. Consequently, we can calculate the mean value and variance of $s_{i}$.

#### 3.5.2. Analysis of Dendritic Layer Output

We analyze the dendritic layer output, *d*. Since the synaptic outputs are multiplied to obtain the dendritic layer output, the expected value, mean value, and variance of the dendritic layer output, *d*, can be calculated given the distribution of $s_{i}$. As we have derived the expected value of $s_{i}$, we can calculate the expected value of the dendritic layer output, *d*, as follows:

(32) $$E\left[d\right]=\prod_{i=1}^{n}E\left[s_{i}\right]$$

Similarly, the mean value and variance of *d* can be derived from the distribution of $s_{i}$.

#### 3.5.3. Gradient Vanishing Analysis

The gradient of the output neuron with respect to weight, $w_{i}$, is as follows:

(33) $$\frac{\partial o}{\partial w_{i}}=\frac{ke^{-kw_{d}}}{{\left(1+e^{-kw_{d}}\right)}^{2}}\prod_{j\neq i}^{n}s_{j}\frac{kx_{i}e^{-k(w_{i}x_{i}-\theta_{i})}}{{\left(1+e^{-k(w_{i}x_{i}-\theta_{i})}\right)}^{2}}$$

To analyze the gradient vanishing problem, we need to consider the magnitude of a gradient. Since the gradient is a product of terms involving the synaptic outputs, $s_{i}$, we need to ensure that the magnitudes of these terms do not become too small, which would cause the gradient to vanish. From our derived expected values, mean values, and variances of the synaptic outputs and dendritic layer output, we can see that the non-negative initialization method yields a balanced distribution of synaptic outputs, $s_{i}$. This balance helps prevent the magnitudes of the terms in the gradient from becoming too small, thus alleviating the gradient vanishing problem. Moreover, since the weights are initialized within the range of [0,b], the influence of negative weights causing a gradient vanishing problem is eliminated. In summary, the non-negative initialization method, which initializes the weights within the range of [0,b], successfully addresses the gradient vanishing problem. Through a thorough mathematical analysis of the expected values, mean values, and variances of the synaptic outputs and dendritic layer output, we demonstrated the effectiveness of this initialization method in mitigating the gradient vanishing problem.

Each symbol used throughout our equations and mathematical formulations is listed along with a detailed description in Table 1. This table is intended to assist readers in understanding the mathematical models and analyses more intuitively, preventing any ambiguity that might arise from the use of symbols.

## 4. Experimental Results

### 4.1. Experimental Setup

In this study, we evaluate the performance of various weight initialization methods for DNM on four different UCI datasets: Ionosphere (D1), Parkinson’s (D2), Sonar (D3), and Vertebral Column (D4), whose data have been collected by various sophisticated sensors and devices. The experimental setup is designed to ensure a comprehensive analysis of DNM’s performance under various initialization conditions. In particular, these four datasets are chosen to represent classification tasks with various levels of data dimensions, from 6 features in the Vertebral Column dataset (low dimension) to 60 features in the Sonar dataset (relatively high dimension). The main characteristics of the four UCI datasets are summarized in Table 2, and the main parameters of DNM are provided in Table 3. To ensure the reproducibility of our experiments and provide clarity on the experimental setup, we include comprehensive details regarding the software and hardware used in our study. The DNMs were implemented using Python version 3.10, with the deep learning framework PyTorch version 2.0.0. The experiments were executed on a workstation equipped with an Intel Core i9-13900K CPU, 64GB of Kingston RAM, and an NVIDIA GeForce RTX 4090 GPU. All the components, including the CPU, RAM, and GPU, are manufactured by Intel, Kingston, and NVIDIA, respectively, with their headquarters located in the United States.

To ensure a comprehensive evaluation of the model, we randomly split each dataset into 70% training data and 30% testing data. The model was trained for 600 epochs, and we conducted 10 runs for each weight initialization method to account for potential variability in the results.

### 4.2. Evaluation Metrics

The primary goal of classification tasks is to assign objects to one of several predefined categories or classes based on their features. In our experiments, we focus on binary classification problems. To evaluate the performance of DNM and the impact of different initialization methods, we use various evaluation metrics. These metrics help us gain a deeper understanding of the model’s strengths and weaknesses, as well as provide quantitative insights into its performance.

In the realm of classification tasks, accuracy represents a fundamental metric, quantifying the percentage of instances that are classified correctly relative to the entire dataset. Despite its intuitive nature and widespread use, accuracy might not always offer a clear picture of performance, particularly in scenarios with imbalanced datasets where its interpretation could be misleading. Complementing accuracy, learning curves offer critical insight into a model’s performance dynamics in relation to the size of the training set and against increasing training periods. By visualizing how training and validation accuracies evolve through successive epochs, these curves become indispensable tools for detecting overfitting or underfitting trends, providing an understanding of how the model matures with added data.

Furthermore, the receiver operating characteristic (ROC) curve serves as an essential evaluative graph that illustrates a classifier’s discernment capacity at various threshold levels. It contrasts the true positive rate against the false positive rate, revealing the nuanced trade-offs faced when adjusting the classification threshold. The integral of this curve, known as the area under the ROC curve (AUC-ROC), distills the classifier’s overall effectiveness into a singular metric, with a score of 1 symbolizing an impeccable model, while 0.5 denotes no discriminative power—akin to random guessing. Equally pivotal, especially in scenarios of class imbalance, the precision–recall (PR) curve sheds light on the balance between the precision (the positive predictive value) and the recall rate (the true positive rate). This curve directs focus onto the performance concerning the positive class. Analogous to AUC-ROC, the area under the PR curve (AUC-PR) encapsulates the curve’s information into a singular value, indicative of the model’s precision and recall efficacy—with higher scores correlating to superior performance.

Deploying this suite of evaluation metrics allows for a multi-faceted analysis of DNM efficacy. These tools are indispensable for revealing the nuanced impacts of diverse weight initialization techniques on the capabilities of DNMs to navigate and succeed in classification challenges.

### 4.3. Experimental Results

In this section, we present the experimental results obtained by training DNM via the four datasets by using different weight initialization methods.

#### 4.3.1. Loss Curves

**Figure 2:**
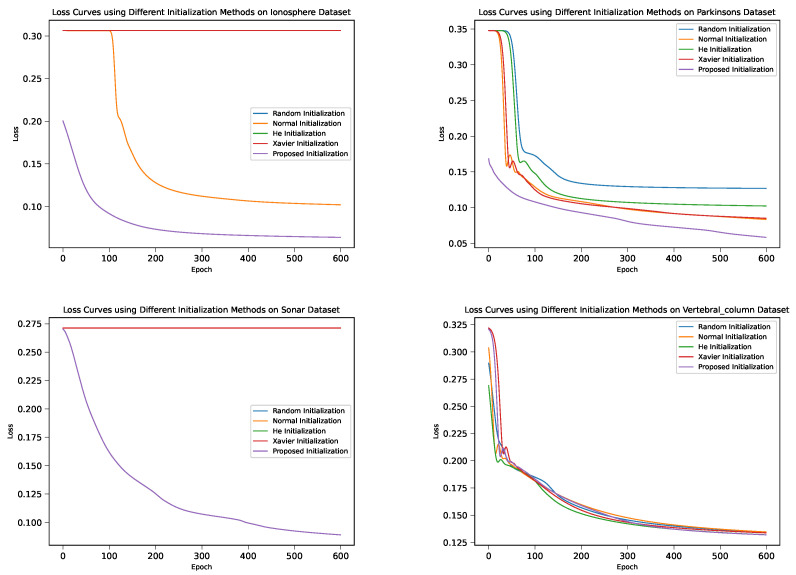
Loss curves for different weight initialization methods in the four datasets.

Figure 2 shows the loss curves for the DNM with different weight initialization methods. The loss curves provide insights into the optimization process and the stability of training. It can be observed that the proposed initialization methods lead to a smoother decrease in the loss values, suggesting more stable training dynamics than its four peers.

#### 4.3.2. Accuracy and Learning Curves

**Figure 3:**
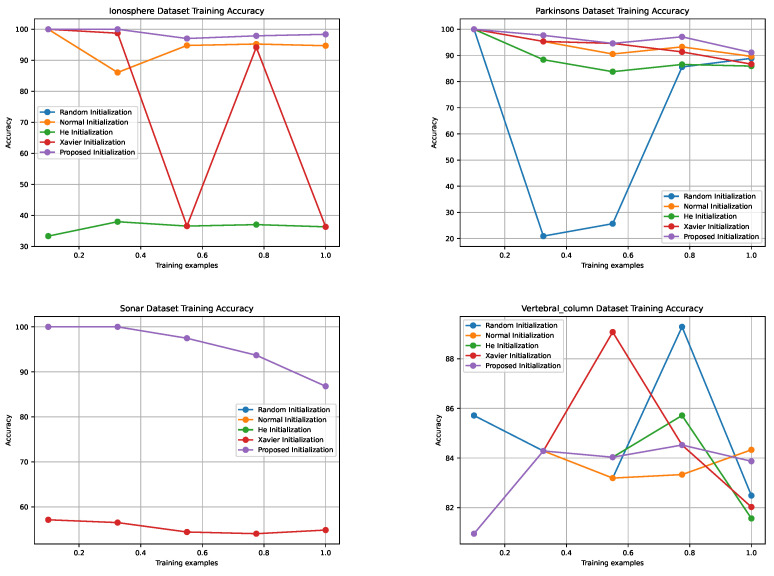
Comparison of Training Accuracies for Different Weight Initialization Methods Across Four Datasets with Varying Training Examples.

**Figure 4:**
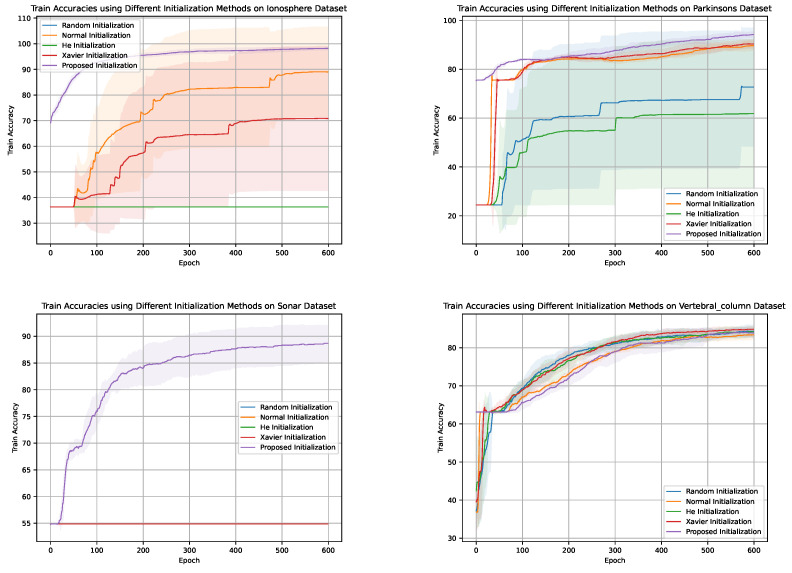
Training accuracies of different weight initialization methods in the four datasets.

**Figure 5:**
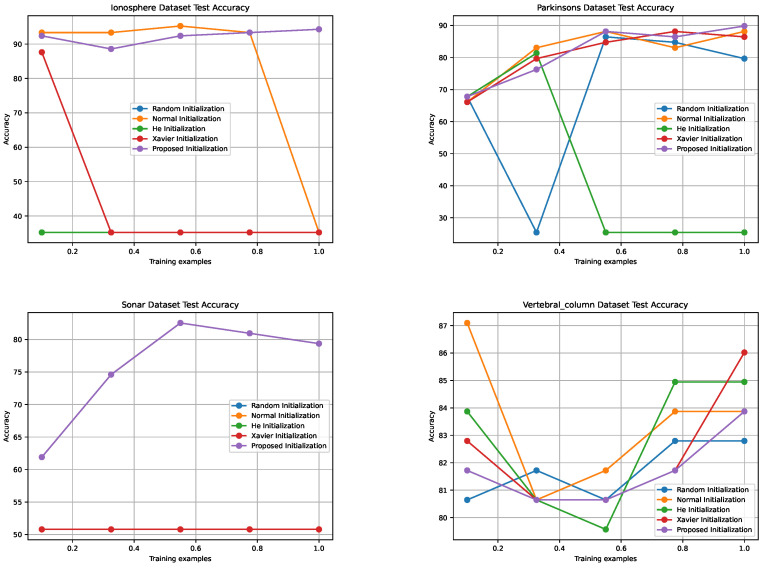
Comparison of Test Accuracies for Different Weight Initialization Methods Across Four Datasets with Varying Training Examples.

**Figure 6:**
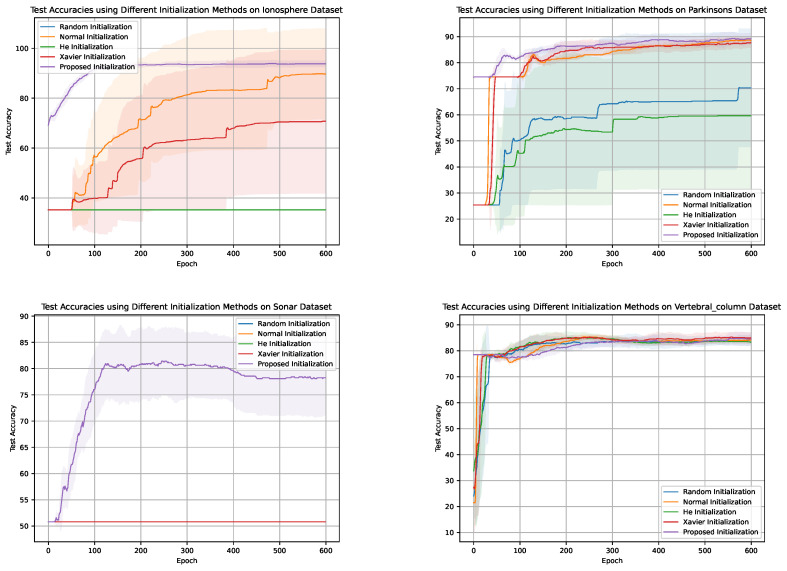
Test accuracies for different weight initialization methods in the four datasets.

Figure 3, Figure 4, Figure 5 and Figure 6 illustrate the learning curves for each weight initialization method in the training and testing datasets. It is evident that different initialization methods have varying impacts on the convergence and final accuracy of DNM. The proposed initialization methods demonstrate the fastest convergence and achieve the highest accuracies, indicating their effectiveness in initializing DNM.

Comparison of convergence speeds for various initialization methods on Dataset D2. The shaded regions denote the 95% confidence intervals around the mean convergence epochs, illustrating the expected range of variability in convergence times

#### 4.3.3. ROC Curves and PR Curves

**Figure 7:**
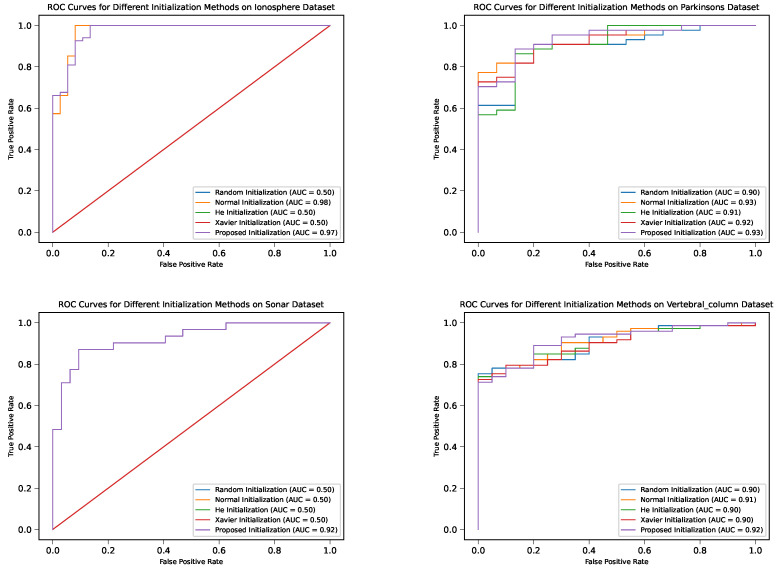
ROC curves for different weight initialization methods in the four datasets.

**Figure 8:**
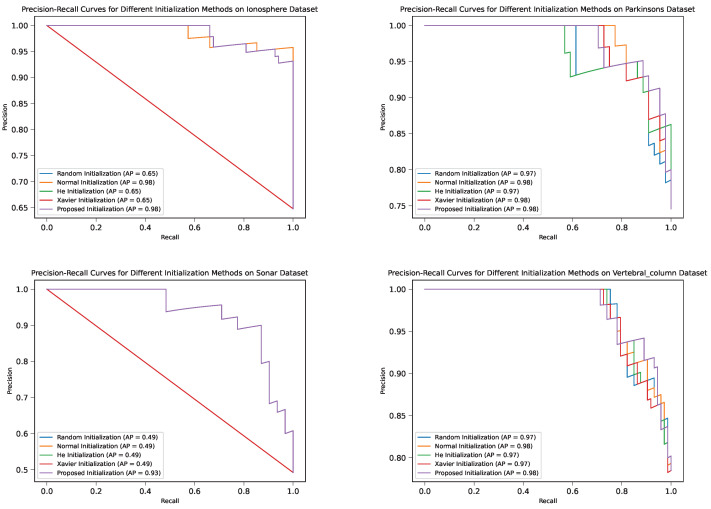
PR curves for different weight initialization methods in the four datasets.

The confusion matrices, ROC curves, and PR curves offer additional insights into the classification performance of DNM with different weight initialization methods. Figure 7 and Figure 8 display these evaluation metrics for each dataset.

These results demonstrate that the proposed initialization methods consistently yield superior performance in terms of classification metrics, such as precision, recall, and F1 score, compared to their peers. This highlights the benefits of employing the proposed initialization techniques in training DNMs for classification tasks.

We also summarized the experimental results in the form of a single comprehensive table, including accuracy, F1 score, recall, precision, and other relevant metrics for each dataset and weight initialization method in Table 4. To ensure the reliability of our experimental findings, we employed statistical tests to assess the significance of the observed differences in performance between our proposed method and the existing methods. Specifically, we use the t-statistic and corresponding p-value to determine whether the differences in mean performance metrics are statistically significant.

T-statistic is a ratio of the departure of an estimated parameter from its hypothesized value to its standard error. It is defined as follows:

(34) $$t=\frac{\bar{X}-\mu }{s/\sqrt{n}}$$

where $\bar{X}$ is the sample mean, μ is the hypothesized population mean, *s* is the sample standard deviation, and *n* is the sample size.

The *p*-value represents the probability of observing a test statistic at least as extreme as the one observed, under the assumption that the null hypothesis is true. A lower *p*-value indicates stronger evidence against the null hypothesis. In our experiments, we considered a *p*-value of less than 0.05 to be statistically significant.

### 4.4. Experimental Results Analysis

In this section, we analyze the experimental results obtained from the four datasets. Our primary focus is on the impact of the proposed initialization methods on the convergence and accuracy of DNM. Building upon the initial analysis of the experimental results, we delve deeper into the performance of the proposed initialization method and highlight several key findings:

1. The proposed initialization method demonstrates consistently high performance across all four datasets. It effectively initializes DNM and enables it to achieve DNM’s full potential, regardless of a dataset’s specific characteristics.
2. For datasets with a larger number of features, such as the Sonar and Ionosphere datasets, the proposed initialization method exhibits superior performance to its peers. This is particularly evident in the Sonar dataset, where our method is the only initialization method that makes the model converge successfully. The results suggest that our method is well-suited for handling high-dimensional datasets and can overcome the challenges posed by complex feature space.
3. From the loss curves, it is evident that our proposed initialization method converges faster than its peers. This indicates that our method not only helps us address the gradient vanishing problem but also facilitates rapid training of DNMs, allowing for efficient model optimization and potentially reducing the overall training time.
4. The experimental results reveal that for datasets with fewer features, all initialization methods perform well in training DNM. This suggests that the impact of the initialization methods is less pronounced in handling lower-dimensional datasets. However, the consistency of our proposed method across diverse datasets still underlines its effectiveness and general applicability.
5. Our proposed initialization method achieves higher accuracy with fewer iterations than its peers. This finding further underscores the efficiency of our methods, as they enable DNMs to reach excellent performance more quickly. In practice, this may result in substantial computational savings, especially in scenarios where rapid model training is crucial.
6. While our method did not achieve the highest accuracy on dataset D4, the results were competitive and closely aligned with the best-reported outcome. Such a finding is typical in machine learning research, where no single method consistently outperforms others across all datasets. The slight variance in performance can be attributed to the unique characteristics of dataset D4, as well as the randomness in the training process inherent to neural networks.

In summary, the extended analysis of the experimental results reinforces the value of our proposed initialization method in addressing a gradient vanishing problem, accelerating a convergence process, and enhancing the overall performance of DNMs across various datasets. The versatility and robustness of our method make it a valuable addition to the repertoire of weight initialization techniques for deep neural networks.

## 5. Conclusions and Future Directions

In this study, we developed a novel and practical initialization technique tailored specifically for DNMs. This straightforward and effective method leverages the unique characteristics of DNMs to enhance their training dynamics and convergence potential. Our comprehensive experiments, utilizing a range of benchmark datasets with varying dimensional complexities, highlighted the method’s superior performance, especially in high-dimensional contexts where it effectively combats the issue of gradient vanishing. Notably, in the Sonar dataset featuring 60 attributes, our method is the only one to achieve successful model convergence. Our experiments on various datasets (D1–D4) reveal the proposed method’s robustness and efficacy. For instance, in the Ionosphere dataset (D1), our method achieves an impressive test accuracy of 94% with a standard deviation of 0.01, outperforming other methods significantly. Similarly, in the Parkinson’s dataset (D2), we observe a test accuracy of 89%, which is indicative of the method’s reliability. Even in cases where our method achieves the highest scores, such as in the Vertebral Column dataset (D4), it delivers competitive results with a test accuracy of 85% ± 3% and a test precision of 90% ± 1%, closely trailing the best-performing method, with its corresponding value of 85% ± 2% and 90% ± 1%. These quantitative outcomes underscore the proposed method’s capacity to maintain high performance across varying high-dimensional datasets and validate its potential as a reliable initialization strategy for DNMs.

Looking forward, the applicability of our initialization method is poised to broaden significantly, encompassing the evolving and complex domains of DNM applications. Recent studies such as those by Liu et al. [45], Zhang et al. [46], and Ding et al. [47] have expanded the horizon of DNMs into areas of medical segmentation, vision transformers for image recognition, and multi-input–multi-output models. These advancements signify the fertile ground for future research, where our initialization technique could be pivotal in unlocking the full computational prowess of DNMs. We anticipate that our work will act as a catalyst for further research endeavors, contributing to the evolution of neural network initialization methods and enhancing their effectiveness across a diverse array of real-world applications.

## Figures and Tables

**Table 1:** List of symbols used in the equations.

Symbol	Description
*o*	Output neuron
$w_{i}$	Weight associated with the *i*-th input
$k_{s}$	Scaling factor for the soma layer
*k*	Scaling factor for the synaptic layer
$w_{d}$	Weighted sum of dendritic inputs
$\theta_{s}$	Threshold for the soma
$\theta_{i}$	Threshold for the *i*-th synapse
$s_{j}$	Output from the *j*-th synapse
$x_{i}$	*i*-th input value to the synapse

**Table 2:** Characteristics of the UCI datasets.

Dataset	Instances	Attributes	Classes	Task
Ionosphere	351	34	2	Binary Classification
Vertebral column	309	6	2	Binary Classification
Parkinson’s	195	22	2	Binary Classification
Sonar	208	60	2	Binary Classification

**Table 3:** The main parameters and initialization methods.

Method	Parameter	Value
DNM	Activation function	Sigmoid
	Synaptic layer parameter *k*	5
	Soma layer parameter $k_{s}$	2
	Threshold $\theta_{s}$	0.3
	The number of dendrites *M*	8
	Epoch	600

**Table 4:** Comparing the classification performances for different initialization methods (D1–4 corresponds to the Ionosphere, Parkinson’s, Sonar, and Vertebral Column). Bold values indicate the best experimental results among the compared datasets/conditions.

		Train				Test					
Dataset	Method	Accuracy	Precision	F1 Score	Recall	Accuracy	Precision	F1 Score	Recall	t-Stat.	p-Test
D1	Random	0.36 ± 0	0 ± 0	0 ± 0	0 ± 0	0.35 ± 0	0 ± 0	0 ± 0	0 ± 0	135.65	1.51 × 10^−28^
	Normal	0.89 ± 0.18	0.85 ± 0.29	0.86 ± 0.29	0.88 ± 0.3	0.90 ± 0.18	0.85 ± 0.29	0.87 ± 0.29	0.90 ± 0.3	0.67	0.51
	He	0.36 ± 0	0 ± 0	0 ± 0	0 ± 0	0.35 ± 0	0 ± 0	0 ± 0	0 ± 0	135.65	1.51 × 10^−28^
	Xavier	0.71 ± 0.28	0.56 ± 0.46	0.57 ± 0.47	0.59 ± 0.48	0.71 ± 0.29	0.56 ± 0.46	0.58 ± 0.47	0.59 ± 0.49	2.38	0.03
	Proposed	**0.98 ± 0.01**	**0.98 ± 0.01**	**0.99 ± 0.01**	**0.99 ± 0.01**	**0.94 ± 0.01**	**0.92 ± 0.01**	**0.95 ± 0.01**	**0.99 ± 0.01**	-	-
D2	Random	0.73 ± 0.25	0.68 ± 0.35	0.73 ± 0.37	0.78 ± 0.39	0.7 ± 0.23	0.67 ± 0.34	0.71 ± 0.36	0.74 ± 0.38	2.47	0.02
	Normal	0.90 ± 0.02	0.93 ± 0.01	0.93 ± 0.01	0.94 ± 0.03	0.88 ± 0.01	**0.92 ± 0.01**	0.92 ± 0.01	0.92 ± 0.02	0.77	0.44
	He	0.62 ± 0.31	0.52 ± 0.43	0.55 ± 0.45	0.58 ± 0.48	0.6 ± 0.28	0.51 ± 0.42	0.53 ± 0.44	0.55 ± 0.45	3.15	0.01
	Xavier	0.9 ± 0.02	0.93 ± 0.01	0.94 ± 0.01	0.94 ± 0.02	0.88 ± 0.02	0.91 ± 0.03	0.92 ± 0.01	0.93 ± 0.03	1.60	0.13
	Proposed	**0.94 ± 0.01**	**0.95 ± 0.01**	**0.96 ± 0.01**	**0.97 ± 0.01**	**0.89 ± 0.02**	0.91 ± 0.03	**0.93 ± 0.01**	**0.95 ± 0.02**	-	-
D3	Random	0.55 ± 0	0 ± 0	0 ± 0	0 ± 0	0.51 ± 0	0 ± 0	0 ± 0	0 ± 0	11.43	1.10 × 10^−9^
	Normal	0.55 ± 0	0 ± 0	0 ± 0	0 ± 0	0.51 ± 0	0 ± 0	0 ± 0	0 ± 0	11.43	1.10 × 10^−9^
	He	0.55 ± 0	0 ± 0	0 ± 0	0 ± 0	0.51 ± 0	0 ± 0	0 ± 0	0 ± 0	11.43	1.10 × 10^−9^
	Xavier	0.55 ± 0	0 ± 0	0 ± 0	0 ± 0	0.51 ± 0	0 ± 0	0 ± 0	0 ± 0	11.43	1.10 × 10^−9^
	Proposed	**0.89 ± 0.04**	**0.93 ± 0.02**	**0.86 ± 0.05**	**0.81 ± 0.08**	**0.78 ± 0.07**	**0.85 ± 0.07**	**0.75 ± 0.09**	**0.67 ± 0.12**	-	-
D4	Random	0.84 ± 0.02	0.87 ± 0.01	**0.88 ± 0.01**	0.87 ± 0.03	0.84 ± 0.02	**0.91 ± 0.02**	0.90 ± 0.02	0.89 ± 0.04	0.39	0.70
	Normal	0.83 ± 0.01	0.87 ± 0.01	0.87 ± 0.01	0.87 ± 0.02	0.84 ± 0.01	0.89 ± 0.01	**0.90 ± 0.01**	0.90 ± 0.01	0.47	0.64
	He	0.84 ± 0.01	0.87 ± 0.01	**0.88 ± 0.01**	0.87 ± 0.03	0.83 ± 0.01	0.90 ± 0.01	0.89 ± 0	0.89 ± 0.01	1.34	0.20
	Xavier	**0.85 ± 0.01**	**0.88 ± 0.01**	0.87 ± 0.01	**0.89 ± 0.02**	**0.85 ± 0.02**	0.90 ± 0.01	**0.90 ± 0.01**	**0.91 ± 0.03**	−0.38	0.70
	Proposed	0.84 ± 0.01	0.87 ± 0.01	0.87 ± 0.02	0.88 ± 0.04	0.85 ± 0.03	0.90 ± 0.01	0.90 ± 0.02	0.90 ± 0.03	-	-

## Data Availability

The data utilized in this article were sourced exclusively from the open-access datasets provided by UCI. These datasets are freely available to the public, facilitating a wide range of research and analysis. For ease of access and transparency, the specific UCI datasets utilized in this study can be found at the following link, accessed on 10 November 2023: https://archive.ics.uci.edu/. We acknowledge and appreciate the contributions of UCI in making these valuable resources available to the research community.
